# Screening and Bioinformatics Analysis of IgA Nephropathy Gene Based on GEO Databases

**DOI:** 10.1155/2019/8794013

**Published:** 2019-07-16

**Authors:** Wang Qian, Wang Xiaoyi, Ye Zi

**Affiliations:** ^1^Department of Nephrology, Shanghai Municipal Hospital of Traditional Chinese Medicine, Shanghai University of Traditional Chinese Medicine, Shanghai, China; ^2^Department of Gastroenterology, Shanghai Traditional Chinese Medicine-Integrated Hospital, Shanghai University of Traditional Chinese Medicine, Shanghai, China

## Abstract

**Purpose:**

To identify novel biomarkers of IgA nephropathy (IgAN) through bioinformatics analysis and elucidate the possible molecular mechanism.

**Methods:**

The GSE93798 and GSE73953 datasets containing microarray data from IgAN patients and healthy controls were downloaded from the GEO database and analyzed by the GEO2R web tool to obtain different expressed genes (DEGs). Gene Ontology (GO), Kyoto Encyclopedia of Genes and Genomes (KEGG) enrichment analysis, protein-protein interaction (PPI), and Biological Networks Gene Oncology tool (BiNGO) were then performed to elucidate the molecular mechanism of IgAN.

**Results:**

A total of 223 DEGs were identified, of which 21 were hub genes, and involved in inflammatory response, cellular response to lipopolysaccharide, transcription factor activity, extracellular exosome, TNF signaling pathway, and MAPK signaling pathway.

**Conclusions:**

TNF and MAPK pathways likely form the basis of IgAN progression, and JUN/JUNB, FOS, NR4A1/2, EGR1, and FOSL1/2 are novel prognostic biomarkers of IgAN.

## 1. Introduction

Immunoglobulin A nephropathy (IgAN) is the most commonly occurring primary glomerulonephritis worldwide and is characterized by increased IgA circulation and deposition in the mesangium [1, 2]. Studies have also shown a genetic predisposition to IgAN [3], with several genes like those encoding transforming growth factor-*β*(TGF-*β*)[4], Megsin [5], Tank binding kinase 1(TBK1) [6], etc. mutated and/or abnormally expressed during IgAN development, and correlated with its prognosis. IgAN is one of the main causes of end-stage renal disease and has diverse clinical manifestations that differ widely in terms of pathological types and prognosis. However, due to the lack of effective diagnostic methods, most IgAN patients are usually diagnosed at the middle and late stages of the disease, leading to poor prognosis. Therefore, it is important to understand the precise molecular mechanisms underlying IgAN in order to develop effective diagnostic and therapeutic strategies. Over the past few decades, microarray technology and bioinformatics analysis have enabled genomic and transcriptomic screening of IgAN samples and helped identify the differentially expressed genes (DEGs) involved in its development and progression. However, due to high false positive rates, it is difficult to obtain reliable results from independent microarrays. Therefore, we analyzed two gene expression microarray datasets from the Gene Expression Omnibus (GEO) database to identify the DEGs between IgAN and normal human tissues. Subsequently, Gene Ontology (GO) and Kyoto Encyclopedia of Genes and Genomes (KEGG) enrichment analysis and protein-protein interaction (PPI) were performed to elucidate the molecular mechanisms of the DEGs. We selected 8 out of 41 hub genes that are potential targets for the diagnosis and treatment of IgAN.

## 2. Materials and Methods

### 2.1. Identification of DEGs

Two microarray datasets of IgAN—GSE93798 and GSE73953—were downloaded from the GEO database (http://www.ncbi.nlm.nih.gov/geo/) using “IgAN” as the search term. GSE93798 was submitted by Liu P, Lassén E et al., and GSE73953 by Okuzaki D, Nojima H et al. GSE93798 was based on Affymetrix's HGU133 Plus 2 chip and includes 42 samples (20 IgAN patients and 22 healthy controls). GSE73953 is based on Agilent's 014850 chip and includes 25 samples (15 IgAN patients and 2 healthy controls). The DEGs between IgAN patients and normal subjects were analyzed using the GEO2R web tool (http://www.ncbi.nlm.nih.gov/geo/geo2r). The screening conditions for the DEGs were absolute value of logFC >1 and adj. P value <0.01.

### 2.2. KEGG and GO Enrichment Analyses of DEGs

The DEGs were uploaded to the DAVID (the database for annotation, visualization, and integrated discovery) version 6.8 (http://david.ncifcrf.gov) [7] online data analysis tool for KEGG and GO analyses. P < 0.05 was considered statistically significant.

### 2.3. PPI Network Construction and Module Analysis

The STRING (version 10.0) (http://string-db.org) tool was used to construct a PPI network of the DEGs with a combined score > 0.4 as the threshold for statistically significant interaction. The Cytoscape (version 3.4.0) software was used to further analyze the interactive network, with the Molecular Complex Detection (MCODE) plugin to identify important molecules in the PPI network. The recognition criteria were MCODE scores > 5, degree cut-off = 2, node score cut-off = 0.2, Max depth = 100, and k-score = 2.

### 2.4. Hub Genes Selection and Analysis

The biological processes of the hub genes were visualized using the Biological Networks Gene Oncology tool (BiNGO) (version 3.0.3) plugin of Cytoscape [8], with significance threshold 0.01 and* Homo sapiens *as the selected organism. Subsequently, the KEGG and GO analyses for the genes in this module were performed using DAVID.

## 3. Results

### 3.1. Identification of DEGs in IgAN

**Figure 1:**
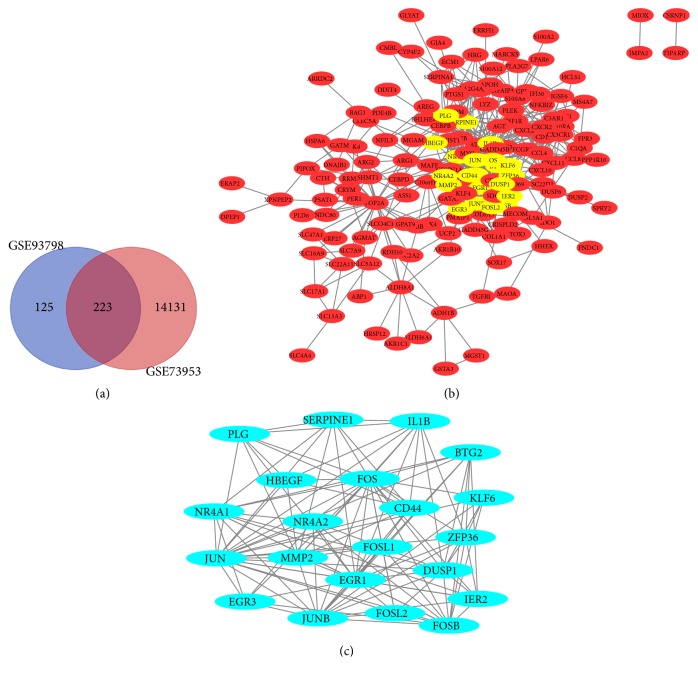
Venn diagram, PPI network, and the most significant module of DEGs. (a) DEGs were selected with absolute value of logFC >1 and adj. P value <0.01 from the GSE93798 and GSE73953 datasets, which showed an overlap of 223 genes. (b) The PPI network of DEGs was constructed using Cytoscape. (c) The most significant module was obtained from PPI network.

**Figure 2:**
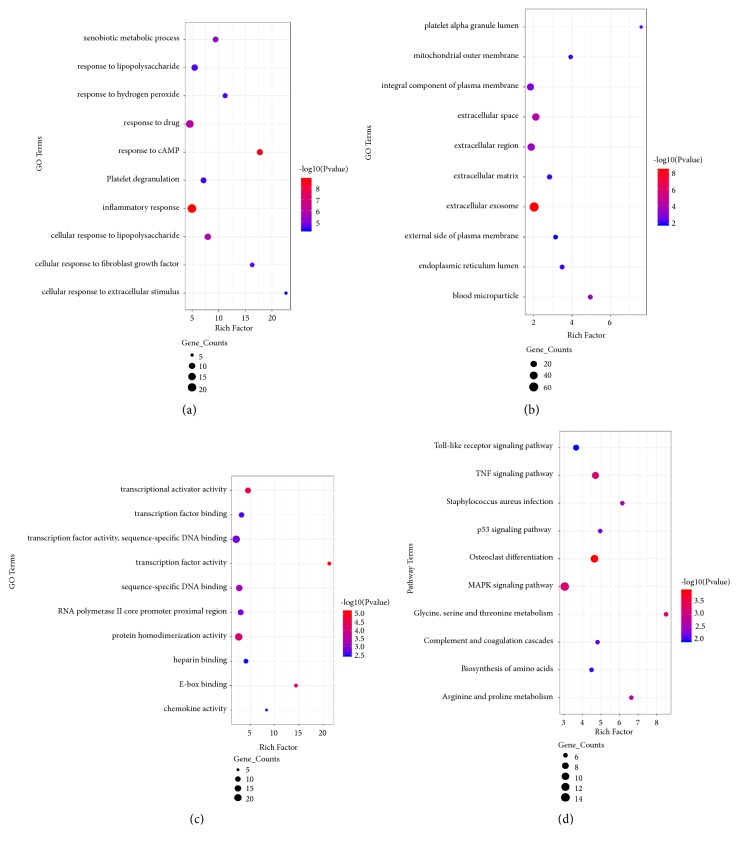
GO and KEGG pathway enrichment analysis of DEGs. The color depth of nodes refers to the P-value. The size of nodes refers to the numbers of genes. (a) GO BP terms. (b) GO CC terms. (c) GO MF terms. (d) KEGG Pathway of DEGs.

After standardizing the microarray results, we identified a total 14353 DEGs in GSE73953 and 348 in GSE93798. A Venn diagram of both datasets showed 223 overlapping genes (Figure 1(a)). GO analysis showed that the biological processes (BP) terms of the DEGs were significantly enriched in inflammatory response, response to cAMP, response to drug, cellular response to lipopolysaccharide, and xenobiotic metabolic process (Figure 2(a)). The molecular function (MF) terms were mainly enriched in transcription factor activity, RNA polymerase II core promoter proximal region sequence-specific binding, transcriptional activator activity, and E-box binding (Figure 2(b)). Finally, cell component (CC) terms were mainly enriched in extracellular exosome, extracellular space, extracellular region, and blood microparticles (Figure 2(c)). KEGG pathway analysis revealed that the downregulated DEGs were mainly enriched in the osteoclast differentiation, TNF signaling, glycine, serine and threonine metabolism, and the MAPK signaling pathway (Figure 2(d)).

### 3.2. PPI Network Construction and Module Analysis

The PPI network of the DEGs was constructed (Figure 1(b)) and the most significant module was obtained using Cytoscape (Figure 1(c)). GO analysis of the module showed significant enrichment in the BP terms of inflammatory response, cellular response to calcium ion, skeletal muscle cell differentiation, cellular response to extracellular stimulus, negative regulation of transcription from RNA polymerase II promoter, and cellular response to corticotropin-releasing hormone stimulus (Figure 4(a)), MF terms of transcription factor activity, RNA polymerase II core promoter proximal region sequence-specific binding, transcriptional activator activity, and RNA polymerase II core promoter proximal region sequence-specific DNA binding MF terms (Figure 4(b)), and CC terms of transcription factor complex, nucleoplasm, and nucleus (Figure 4(c)). KEGG pathway analysis revealed that the downregulated DEGs were mainly enriched in osteoclast differentiation, estrogen signaling, TNF signaling, and MAPK signaling pathways (Figure 4(d)).

### 3.3. Hub Gene Selection and Analysis

**Figure 3:**
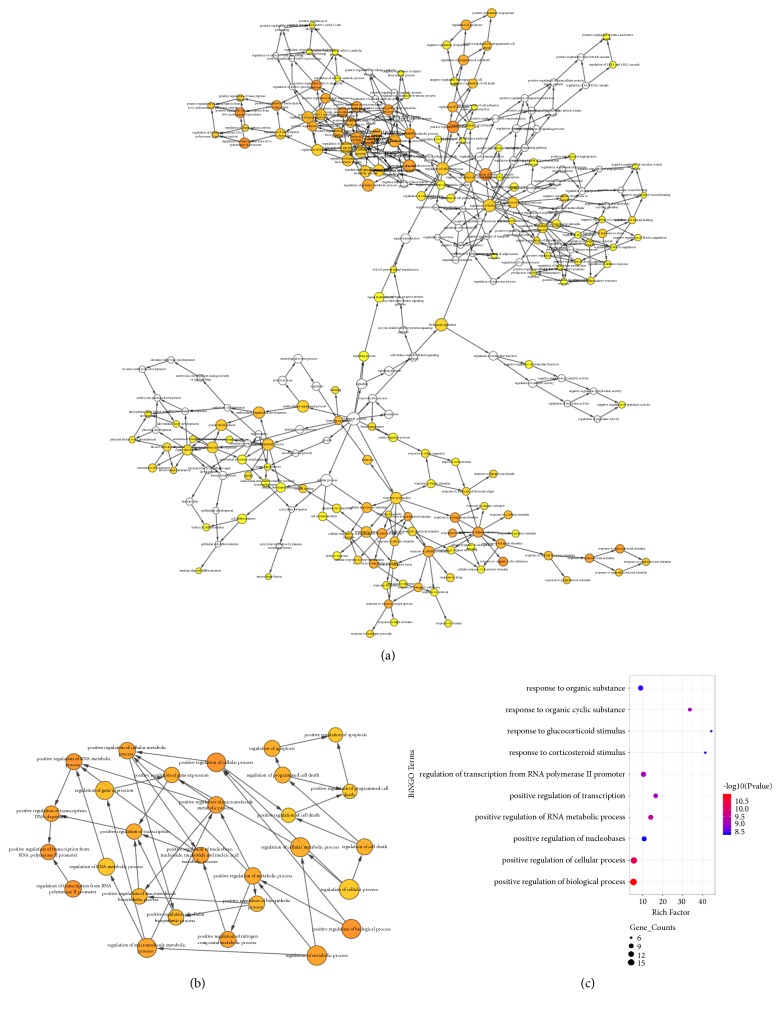
Interaction network and biological process analysis of the hub genes. The color depth of nodes refers to the P-value. The size of nodes refers to the numbers of genes. (a) Hub genes and their coexpression genes were analyzed using BiNGO. (b), (c) The most significant coexpression was obtained from BiNGO network.

**Figure 4:**
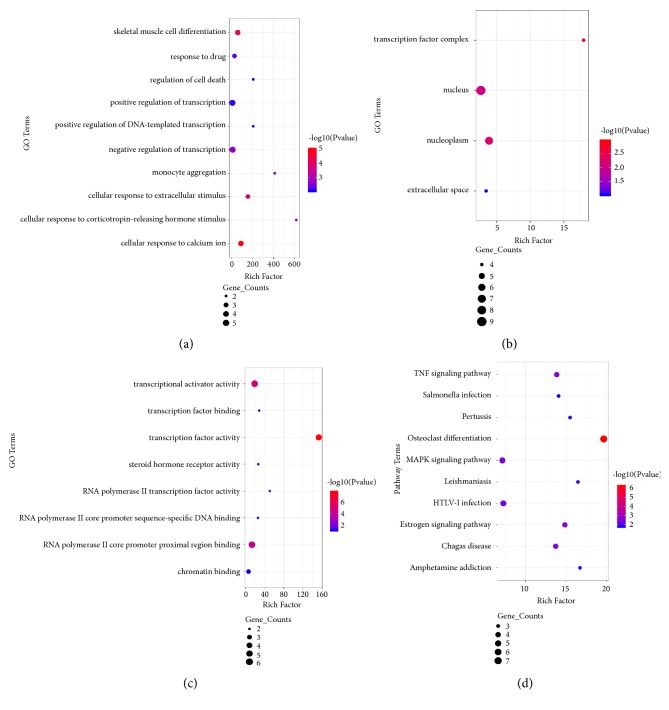
GO and KEGG pathway enrichment analysis of hub genes. The color depth of nodes refers to the P-value. The size of nodes refers to the numbers of genes. (a) GO BP terms. (b) GO CC terms. (c) GO MF terms. (d) KEGG Pathway of hub genes.

A total of 21 genes were identified as hub genes, and their names and MCODE scores are shown in Table 1. A network of the hub genes and their coexpression genes was analyzed using BiNGO tool of Cytoscape (Figure 3(a)), and the significant coexpression genes are shown in Figures 3(b) and 3(c).

## 4. Discussion

IgAN accounts for ~20% to 47% of primary glomerular diseases and is mainly characterized by hematuria, proteinuria, hypertension, and renal dysfunction [9]. The incidence of the disease has been increasing annually, and 30% to 40% of the patients progress to end-stage renal disease (ESRD), which is the main cause of primary glomerulonephritis for renal replacement therapy in China, within 10 years [3]. Although the most common clinical manifestation of IgAN is hematuria, there is considerable heterogeneity among different cases, which makes early diagnosis challenging. We analyzed two IgAN microarray datasets from the GEO database to screen for DEGs in IgAN and identify potential biomarkers. We obtained a total of 223 DEGs, and the upregulated genes were mainly enriched in inflammatory response, cell fibrosis, TNF signaling pathway, and MAPK signaling pathway. Previous studies have shown a significant association of IgAN development and prognosis with the inflammatory reaction [10, 11] and glomerular and tubular fibrosis [12, 13]. Tumor necrosis factor (TNF) is a critical cytokine involved in apoptosis, cell survival, inflammation, and immunity. The MAPK pathway is also associated with tubule-interstitial fibrosis in IgAN [14]. Signaling through the TNF receptor (TNFR1) activates a number of genes via two distinct pathways: NF-kB and the MAPK cascade. Thus, both pathways obtained by enrichment analysis are correlated and involved in the pathogenesis of IgAN.

Using STRING and MCODE, we selected 21 DEGs as the hub genes and found the maximal correlation between JUN/JUNB, FOS, NR4A1/2, EGR1, and FOSL1/2. The expression levels of JUN, an early responding transcription factor, are very low in under normal conditions. Upon receiving an external stimulus, it is rapidly activated and dimerizes with either another JUN protein or with AP-1 to form the FOS protein. The JUNB gene is structurally and functionally very similar to JUN and along with JUN and FOS forms the upstream element of the TNF/TNFR1 pathway. The interaction between the JNK/MAPK and TNF/TNFR1 pathways forms the molecular basis of glomerular and renal interstitial atrophy and apoptosis, which leads to progressive renal damage and renal fibrosis [15]. These pathways are also involved in RAAS activation, complement activation, coagulation cascades [16], and inflammation [17].

FOSL1 and FOSL2 are also members of the FOS gene family [18] and overexpressed in various renal diseases like IgAN, lupus nephropathy, and focal glomerular sclerosis, with pivotal roles in glomerular sclerosis and mesangial proliferation. Rastaldi et al. [19] reported abnormal expression of FOSL1/2 in IgAN patients, and Park et al. found that the FOS proteins are also involved in the disappearance of podocyte foot processes [20]. Podocytes maintain the structure and function of the glomerular filtration membrane and prevent proteinuria.

Early growth response factor 1 (Egr1) is a zinc-finger transcription factor expressed across different eukaryotic cells [21]. In humans, it is expressed in various renal cells, including glomerular MCs, endothelial cells, renal tubular fibroblasts, and epithelial cells [22]. Upregulation of Egr1 is associated with renal fibrosis and inflammation, especially in the development of diabetic nephropathy [23–25]. Its role in the development of IgAN is not completely clear.

NR4A1 and NR4A2 are also two early responding genes associated with cancer [26] and chronic inflammatory diseases [27], including that affecting the kidneys. Westbrook et al. showed increased kidney injury and reduced renal function in the NR4A1 knockout mouse model. NR4A1 plays a significant role in kidney injury via immune activation, and enhancing NR4A1 expression or function is a potential therapeutic strategy against kidney disease [28]. Xin et al. showed the possible involvement of NR4A2 in the development of congenital obstructive nephropathy [29]. However, neither protein has been associated with IgAN.

To summarize, we identified several hub genes involved in the pathological changes of IgAN. The FOS family genes are associated with renal inflammation, fibrosis, and podocyte function during the development and progression of IgAN, but the involvement of JUN, JUNB, NR4A1/2, and EGR1 in this disease has not been widely reported. They are directly related to each other and likely mediate the pathological changes in the kidneys of IgAN patients.

## 5. Conclusion

Twenty-one hub genes were identified that are potential prognostic/diagnostic biomarkers of IgAN. Their biological functions and mechanisms of action in IgAN need to be studied further.

## Figures and Tables

**Table 1:** Functional roles of 21 hub genes.

No.	Gene symbol	MCODE Score
1	CD44	8.00
2	FOSB	8.67
3	ZFP36	7.82
4	IER2	7.82
5	FOSL2	7.82
6	MMP2	8.00
7	FOSL1	8.00
8	EGR3	8.00
9	EGR1	8.67
10	BTG2	8.67
11	NR4A1	7.27
12	FOS	8.67
13	HBEGF	7.00
14	JUNB	8.67
15	IL1B	8.00
16	JUN	8.67
17	DUSP1	8.67
18	NR4A2	7.82
19	KLF6	7.00
20	PLG	8.00
21	SERPINE1	8.00

## Data Availability

The data used to support the findings of this study are available from the corresponding author upon request.
